# Impact of Walking on Glycemic Control and Other Cardiovascular Risk Factors in Type 2 Diabetes: A Meta-Analysis

**DOI:** 10.1371/journal.pone.0109767

**Published:** 2014-10-17

**Authors:** Shanhu Qiu, Xue Cai, Uwe Schumann, Martina Velders, Zilin Sun, Jürgen Michael Steinacker

**Affiliations:** 1 Division of Sports and Rehabilitation Medicine, Department of Medicine II, Ulm University, Ulm, Germany; 2 Department of Endocrinology, Zhongda Hospital, Institute of Diabetes, Medical School, Southeast University, Nanjing, P. R. China; University of Chieti, Italy

## Abstract

**Background:**

Walking is the most popular and most preferred exercise among type 2 diabetes patients, yet compelling evidence regarding its beneficial effects on cardiovascular risk factors is still lacking. The aim of this meta-analysis of randomized controlled trials (RCTs) was to evaluate the association between walking and glycemic control and other cardiovascular risk factors in type 2 diabetes patients.

**Methods:**

Three databases were searched up to August 2014. English-language RCTs were eligible for inclusion if they had assessed the walking effects (duration ≥8 weeks) on glycemic control or other cardiovascular risk factors among type 2 diabetes patients. Data were pooled using a random-effects model. Subgroup analyses based on supervision status and meta-regression analyses of variables regarding characteristics of participants and walking were performed to investigate their association with glycemic control.

**Results:**

Eighteen studies involving 20 RCTs (866 participants) were included. Walking significantly decreased glycosylated haemoglobin A1c (HbA1c) by 0.50% (95% confidence intervals [CI]: −0.78% to −0.21%). Supervised walking was associated with a pronounced decrease in HbA1c (WMD −0.58%, 95% CI: −0.93% to −0.23%), whereas non-supervised walking was not. Further subgroup analysis suggested non-supervised walking using motivational strategies is also effective in decreasing HbA1c (WMD −0.53%, 95% CI: −1.05% to −0.02%). Effects of covariates on HbA1c change were generally unclear. For other cardiovascular risk factors, walking significantly reduced body mass index (BMI) and lowered diastolic blood pressure (DBP), but non-significantly lowered systolic blood pressure (SBP), or changed high-density or low-density lipoprotein cholesterol levels.

**Conclusions:**

This meta-analysis supports that walking decreases HbA1c among type 2 diabetes patients. Supervision or the use of motivational strategies should be suggested when prescribed walking to ensure optimal glycemic control. Walking also reduces BMI and lowers DBP, however, it remains insufficient regarding the association of walking with lowered SBP or improved lipoprotein profiles.

**Trial Registration:**

PROSPERO CRD42014009515

## Background

Regular exercise is a key element in the management of type 2 diabetes [1]–[4]. Current guidelines and positions recommend that patients with type 2 diabetes should undertake moderate to vigorous aerobic exercise that includes running or bicycling to gain cardiovascular benefits [5], [6]. However, most patients with type 2 diabetes are less likely to perform such high impact exercise because of their impaired tolerance of physical capacity [7], [8] and somewhat hard feelings during the exercise.

Walking, as a typical low impact exercise, is the most popular and most preferred exercise among patients with type 2 diabetes [9], [10]. It can be performed at a variety of speeds with different intensities [11], requires no specific skills or sophisticated preexercise evaluation [6], and has comparatively minimal adverse effects [12]. Although previous meta-analyses noted that walking could improve several known risk factors for cardiovascular disease such as blood pressure [13], body mass index (BMI) [14], and high-density lipoprotein cholesterol (HDL-C) [15], none of them had investigated the effects of walking on glycemic control, which is considered the mainstay of type 2 diabetes management. Besides, it should keep in mind that those analyses were conducted mainly on sedentary but healthy adults, conclusions of which cannot be generalized to patients with type 2 diabetes, who are often more unwilling to exercise [16]. Moreover, there exists a large body of evidence that walking interventions can be very successfully implemented in patients with type 2 diabetes [17]–[34], but inconsistent results have been shown with regard to their beneficial effects on health outcomes, such as glycemic control [20], [25], [30], [32], weight reduction [20], [21], blood pressure [19], [20] and lipoprotein profiles [19], [22], questioning whether walking is the best medicine for diabetes [35].

Additionally, supervision is strongly recommended to optimize the exercise training effects on glycemic control [6], [22]. Yet supervision is not always feasible in the primary-care exercise implementation due to the limited and unevenly distributed medical care resources [36]. Moreover, its necessity has been greatly challenged since several studies have pointed out that exercise such as walking in the free-living environment without supervision is also effective in improving glycemic control [18], [20], [23].

Therefore, the primary aim of this meta-analysis of randomized controlled trials (RCTs) was to examine the association of walking with glycemic control, and other cardiovascular risk factors including weight reduction, blood pressure, and lipoprotein profiles among patients with type 2 diabetes. The second aim was to evaluate whether supervised walking would lead to better improvement in glycemic control versus non-supervised walking among patients with type 2 diabetes.

## Methods

### Data sources and searches

The following databases were searched for primary articles: PubMed (from January 1, 1966 to August 8, 2014), the Cochrane Central Register of Controlled Trials (from January 1, 1966 to August 8, 2014) and Web of Science (from January 1, 1945 to August 8, 2014). The initial computer-based search strategies comprised common text words and Medical Subject Heading terms related to exercise, walking and type 2 diabetes, as well as entry terms associated with a highly sensitive search filter for RCTs. Searches were limited to human beings and the language was restricted to English. The reference lists of relevant systematic review/meta-analysis were hand-searched to find other potentially suitable studies. The complete search strategies are shown in Table S1. This meta-analysis is reported according to the Preferred Reporting Items for Systematic Reviews and Meta-Analyses (PRISMA) statement (Checklist S1), and adheres to a registered protocol (PROSPERO CRD42014009515; Table S2).

### Study selection

Studies were eligible for inclusion if they fulfilled the following criteria: (i) enrolled participants diagnosed with type 2 diabetes; (ii) engaged in a structured walking programme; (iii) compared with a control group that received no walking training, but could maintain normal lifestyle or receive usual care; (iv) reported sufficient data to allow calculation of weighted mean difference (WMD) together with 95% confidence intervals (CIs) in the primary outcome – glycemic control as assessed by glycosylated haemoglobin A1c (HbA1c), or the secondary outcomes – weight reduction as indicated by BMI, blood pressure as measured by systolic blood pressure (SBP) or diastolic blood pressure (DBP), or lipoprotein profiles as determined by HDL-C or low-density lipoprotein cholesterol (LDL-C); (v) had a randomized, controlled design. Since the outcome of interest, HbA1c, reflects the average blood glucose concentration during the preceding 8–12 weeks, analyses were limited to studies in which the walking intervention lasted at least 8 weeks.

Studies were excluded if they (i) included participants diagnosed with pre-diabetes, gestational diabetes, or type 1 diabetes; (ii) had multiple exercise interventions that mixed/combined with other forms/modes of exercise, utilized interventions consisting only of recommending increased daily walking steps by motivational tools or lasting less than 8 weeks, or combined with dietary intervention; (iii) compared with the controls that received regular exercise training; (iv) reported only categorical data of outcomes; or (v) were non-randomized studies, posters or just abstracts. Studies that gave insufficient information regarding the forms of aerobic exercise interventions were also excluded if the related information could not be obtained from the corresponding authors.

### Data extraction and quality assessment

Initial screen was based on titles or abstracts of retrieved publications; if they provided inadequate information with regard to inclusion or exclusion criteria, full-text articles were retrieved and evaluated. For each study, data regarding study sources (including author and publication year), characteristics of study population (including sample size, baseline mean age, BMI, sex [proportion of females] and duration of diabetes), characteristics of walking interventions (including frequency, intensity, time of each bout, length of intervention, and supervision status [that is, with or without]), outcomes (including at least one of the followings: HbA1c, BMI, DBP, SBP, HDL-C and LDL-C), adherence and dropout rates, were extracted.

The methodological quality of each eligible study was assessed using the Cochrane Collaboration’s risk of bias tool [37], which includes random sequence generation, allocation concealment, blinding of participants and personnel, blinding of outcome assessment, incomplete outcome data and selective reporting. Risk of bias for each item was judged as low, unclear or high, based on the criteria in the Cochrane Handbook for Systematic Reviews [37].

Two authors (S.Q. and X.C.) independently performed the literature selection, data collection, and quality assessment. Discrepancies on the inclusion of studies or quality assessment were solved by consensus or discussion.

### Data synthesis and analysis

For studies that reported standard error of mean, the standard deviation was obtained by multiplying by the square root of the corresponding sample size. For studies that compared 2 different walking interventions with a single control group, the “shared” group was split into 2 different groups with weighted smaller sample sizes in relation to different walking interventions. This was applied to give reasonably independent comparisons and to overcome a unit-of-analysis error [37]. For studies that gave outcomes at more than one time point during the intervention, data from the last time point were used for primary analyses.

Change scores from baseline or final values of each outcome variable were entered in the same meta-analysis, as suggested in the Cochrane Handbook for Systematic Reviews [37]. Data from intention-to-treat or per-protocol analyses were entered when available. The heterogeneity among studies was assessed using Cochran Q test, with a *P* value of <.10 being considered of statistical significance. The degree of inconsistency across studies due to heterogeneity was determined using *I^2^* statistic, where an *I^2^* value ≥50% represented substantial heterogeneity. To account for between-study heterogeneity, the pooled-effect estimates expressed as WMD and the corresponding 95% CIs of each outcome were calculated using a random-effects model.

Subgroup analyses were performed to investigate the differences in outcome estimates across studies on the basis of supervision status. Univariate, weighted meta-regression analyses were conducted to determine whether the changes in outcome estimates were mediated by the characteristics of participants (baseline mean age [logarithmic transformation] BMI, sex and duration of diabetes) or walking interventions (length of intervention, frequency and volume [frequency × time of each bout]). The above analyses were conducted mainly on the primary outcome. Sensitivity analyses were used to assess the robustness of outcome estimates by removing each trial individually. Publication bias in the meta-analyses was detected and assessed by the Begg's test and Egger's test. Statistical analyses were performed using STATA Software (Version 12.0, College Station, Texas, USA).

## Results

**Figure 1:**
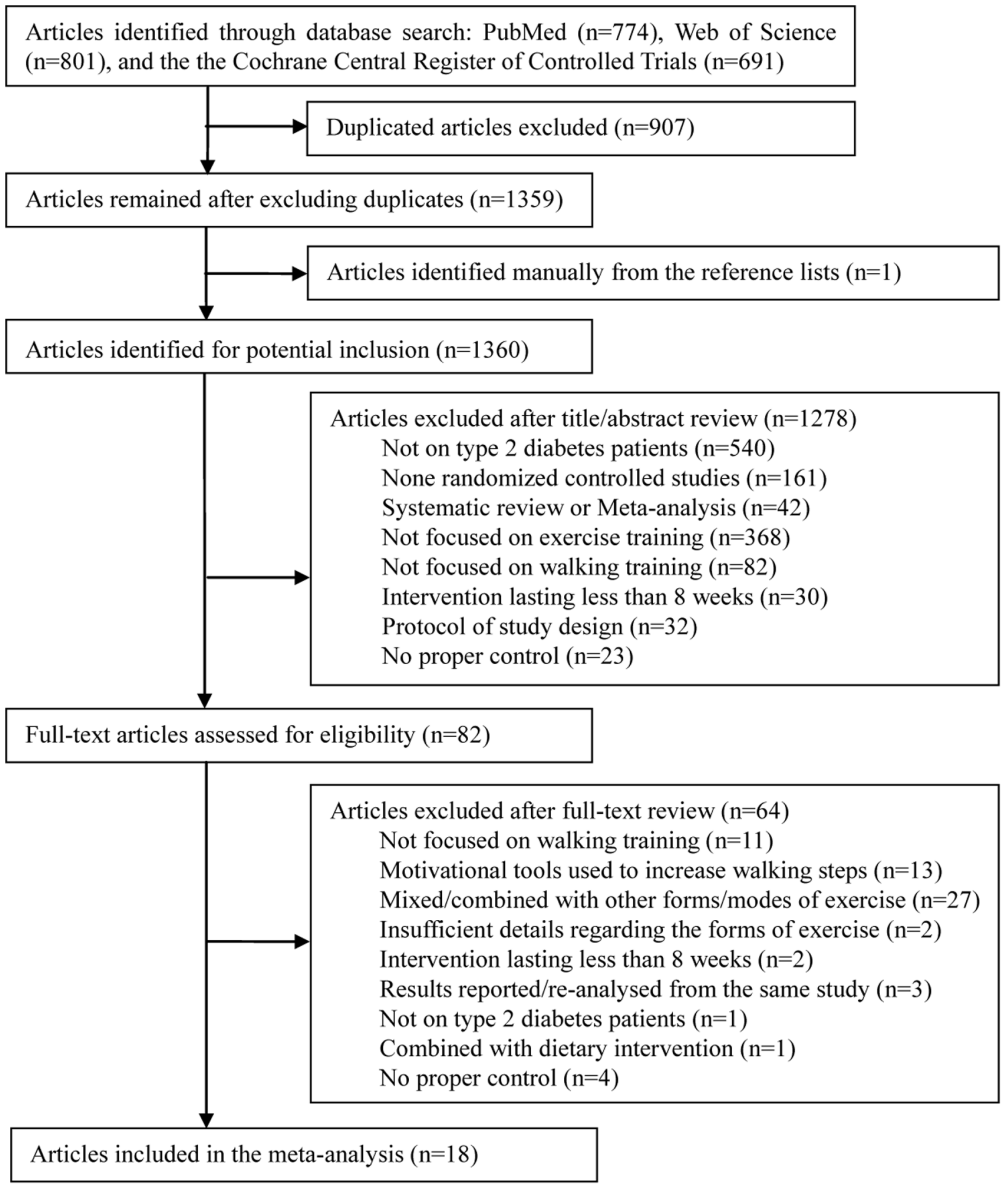
Flow diagram of search and selection processes.

The flow diagram of literature search and study selection is shown in Figure 1. The initial search identified 2266 potentially suitable articles. One additional article was identified by analyzing the reference lists of relevant systematic review/meta-analysis papers searched. After careful screening and independent selection, 18 studies met all inclusion criteria. Among them, 2 studies had 2 different walking groups [20], [34]; therefore, 20 trials were included in the final meta-analysis.

The detailed characteristics of these trials are summarized in Table 1. Of these 20 trials, a total of 866 participants were included, with sample sizes ranging from 16 to 149 in individual trials. All the participants were generally overweight or obese, with the baseline mean BMI ranging from 25.6 kg/m^2^ to 32.7 kg/m^2^. The walking structures varied among trials, with the length of interventions ranging from 8 weeks to 36 weeks. The time of each walking bout ranged from 20 minutes to 120 minutes, and the frequency differed from 3 times/week to 7 times/week, except 1 trial without indication [29]. The intensity of walking was moderate in general, except 3 trials without specification [18], [19], [27]. Eleven trials were conducted under supervision by qualified trainers, while among the remaining 9 trials that conducted without supervision, 5 of them had adopted strategies for promoting the training - that is, 1 had peer support [18] and the other 4 used step counters [20], [23], [32]. Three of the 20 trials were carried out in North America (U.S.) [18], [21], [30], 1 in South America (Brazil) [17], 11 in Asia (4 in South-Korea [24], [26], [32], [33], 3 in India [23], [27], [31], 1 in Iran [28], 1 in Turkey [21] and 2 in Thailand [34]), 4 in Europe (3 in Denmark [19], [20] and 1 in Italy [22]), and 1 in South Africa [25].

**Table 1:** Characteristics of included randomized controlled trials.

Source	Age, year#	No. of subjects	Intervention and control description§	Duration$, weeks	Status of supervision	Adherence,%	Dropouts, %
Belli *et al.* 2011 [17]	53.4 (2.3)	9	Intervention: I: individual ventilatory threshold; F: 3 times/week; T: progressed from 20 minutes at week 1 to 60 minutes at week 5, and then maintained	12	With	92	25
	55.9 (2.2)	10	Control: continued normal lives without systematic exercise				16.7
Goldhaber-Fiebert *et al.* 2003 [18]	60 (10)	33	Intervention: I: not stated; F: 3 times/week; T: 60 minutes	12	Without	Not stated	17.5
	57 (9)	28	Control: continued normal lives without systematic exercise				20
Gram *et al*. 2010 [19]	62 (10)	22	Intervention: I: >40% of VO_2max_; F: 3 times/week; T: at least 30 minutes	16	With	63.5	4.5
	61 (10)	22	Control: continued habitual lifestyle and advised to exercise				0
Karstoft *et al.* 2013a* [20]	60.8 (2.2)	12	Intervention: I: 55% of peak energy-expenditure rate; F: 5 times/week; T: 60 minutes (in total)	16	Without	89	8.3
	57.1 (3.0)	4	Control: continued habitual lifestyle				0
Karstoft *et al.* 2013b* [20]	57.5 (2.4)	12	Intervention: I: 70% of peak energy-expenditure rate; F: 5 times/week; T: 60 minutes (in total)	16	Without	89	8.3
	57.1 (3.0)	4	Control: continued habitual lifestyle				0
Kurban *et al.* 2011 [21]	53.77 (8.2)	30	Intervention: I: moderate; F: 3 times/week; T: 30 minutes	12	With	Not stated	0
	53.57 (6.6)	30	Control: continued habitual lifestyle				0
Negri *et al.* 2010 [22]	65.7 (4.9)	21	Intervention: I: increased gradually from low to moderate; F: 3 times/week; T: 45 minutes	16	With	60	20.5
	65.7 (5.2)	20	Control: continued habitual lifestyle and encouraged to exercise				4.8
Shenoy *et al*. 2010 [23]	53.15 (4.4)	20	Intervention: I: 50–70% of maximum heart rate; F: 5 times/week; T: 150 minutes/week (in total)	8	Without	Not stated	0
	51 (5.4)	20	Control: received no training				0
Sung *et al.* 2012 [24]	70.2 (4.7)	22	Intervention: I: 55–75% of maximum heart rate; F: 3 times/week; T: 30 minutes (1–4 weeks), 35 minutes (5–14 weeks) and 40 minutes (15–24 weeks)	24	With	Not stated	4.8 (in total)
	70.1 (3.6)	18	Control: received usual care				
van Rooijen *et al.* 2004 [25]	54	75	Intervention: I: moderate (12–14 PRE); F: ≥5 times/week; T: progressed from 10 to 45 minutes	12	Without	Not stated	6.3
	55	74	Control: received usual care without exercise				3.9
Ku *et al.* 2010 [26]	55.7 (7.0)	15	Intervention: I: moderate (3.6–5.2 METs); F: 5 times/week; T: 60 minutes	12	With	Not stated	0
	57.8 (8.1)	16	Control: continued habitual lifestyle				0
Arora *et al.* 2009 [27]	52.2 (9.3)	10	Intervention: I: not stated; F: 3 times/week; T: 30 minutes	8	Without	Not stated	0
	58.4 (1.8)	10	Control: continued habitual lifestyle				0
Moghadasi *et al*. 2013 [28]	43 (overall)	8	Intervention: I: 40–59% of VO_2max_; F: 4 times/week; T: 30 minutes (for 2 miles)	12	With	Not stated	0
		8	Control: continued habitual lifestyle				0
Kaplan *et al.* 1985 [29]	54 (overall)	18	Intervention: I: 60–70% of maximal work capacity; F: not stated; T: progressed to 40–60 minutes	10	Without	Not stated	7.4 (in total)
		15	Control: received usual care				
Church *et al*. 2010 [30]	53.7 (9.1)	72	Intervention: I: about 65% of VO_2max_; F: 3 times/week; T: 140 minutes/week (in total)	36	With	>70, for the most	4.2
	58.6 (8.2)	41	Control: continued normal lives without systematic exercise				9.7
Dixit *et al*. 2014 [31]	54.4 (1.2)	29	Intervention: I: 12–13 PRE; F: ≥3 times/week; T: ≥150 minutes/week (in total)	8	With	Not stated	27.5
	59.5 (1.2)	37	Control: received usual care				21.3
Koo *et al.* 2010 [32]	59 (4)	13	Intervention: I: moderate-to-vigorous; F: 7 times/week; T: 120 minutes	12	Without	≥80	0
	57 (8)	18	Control: received usual care				0
Kwon *et al*. 2010 [33]	55.5 (7.5)	13	Intervention: I: anaerobic threshold (moderate intensity); F: 5 times/week; T: 60 minutes	12	Without	Not stated	15.6 (in total)
	57.5 (8.6)	14	Control: continued normal lives				
Mitranun *et al*. 2014a** [34]	61.7 (2.7)	14	Intervention: I: 60–65% of VO2peak; F: 3 times/week; T: 30 minutes	12	With	≥80	6.7
	60.9 (2.4)	7	Control: continued sedentary lives				0
Mitranun *et al*. 2014b** [34]	61.2 (2.8)	14	Intervention: I: 50–80% of VO2peak; F: 3 times/week; T: 20–30 minutes	12	With	≥80	6.7
	60.9 (2.4)	8	Control: continued sedentary lives				0

I: intensity; F: frequency; T: time of each walking bout; PRE; perceived rate of exertion; VO_2max_, maximal oxygen consumption; METs, metabolic equivalents.

# Age was represented as mean (SD), or mean if SD was not provided, or imputed with a mean.

§ Characteristics of walking training described did not include warm-up or cool-down periods unless indicated.

$ Duration meant length of walking intervention in this meta-analysis.

^*^The same study which included 2 different walking groups: “a” was a continuous walking training group; “b” was an energy expenditure–matched interval-walking training group.

^**^The same study which included 2 different walking groups: “a” was a continuous walking training group; “b” was a total oxygen consumption-matched interval-walking training group.

The risk of bias assessment for each trial is listed in Table S3. Among these 20 trials, 60% (12/20) reported adequate randomization sequence generation, 10% (2/20) provided proper allocation concealment, 75% (15/20) utilized proper methods in dealing with incomplete outcome data, and 100% (20/20) described losses to follow-up and exclusions. Because of the nature of walking intervention, none of the included trials had complete blinding of participants and personnel. Yet the outcome assessment for each trial is not likely to be influenced by lacking of blinding, given the outcome variables of interest were all measured in the standard approaches.

Only 9 of the 20 trials presented data on adherence to the walking intervention, with all the adherence rates more than 60%. Fifteen of the 20 trials reported dropout rates less than 10% (Table 1). No major adverse events related to walking, such as musculoskeletal injury or severe hypoglycemia, were reported; except 3 trials that reported mild hypoglycemia [19], [22], [31].

### Primary outcome

#### Effect on glycemic control

**Figure 2:**
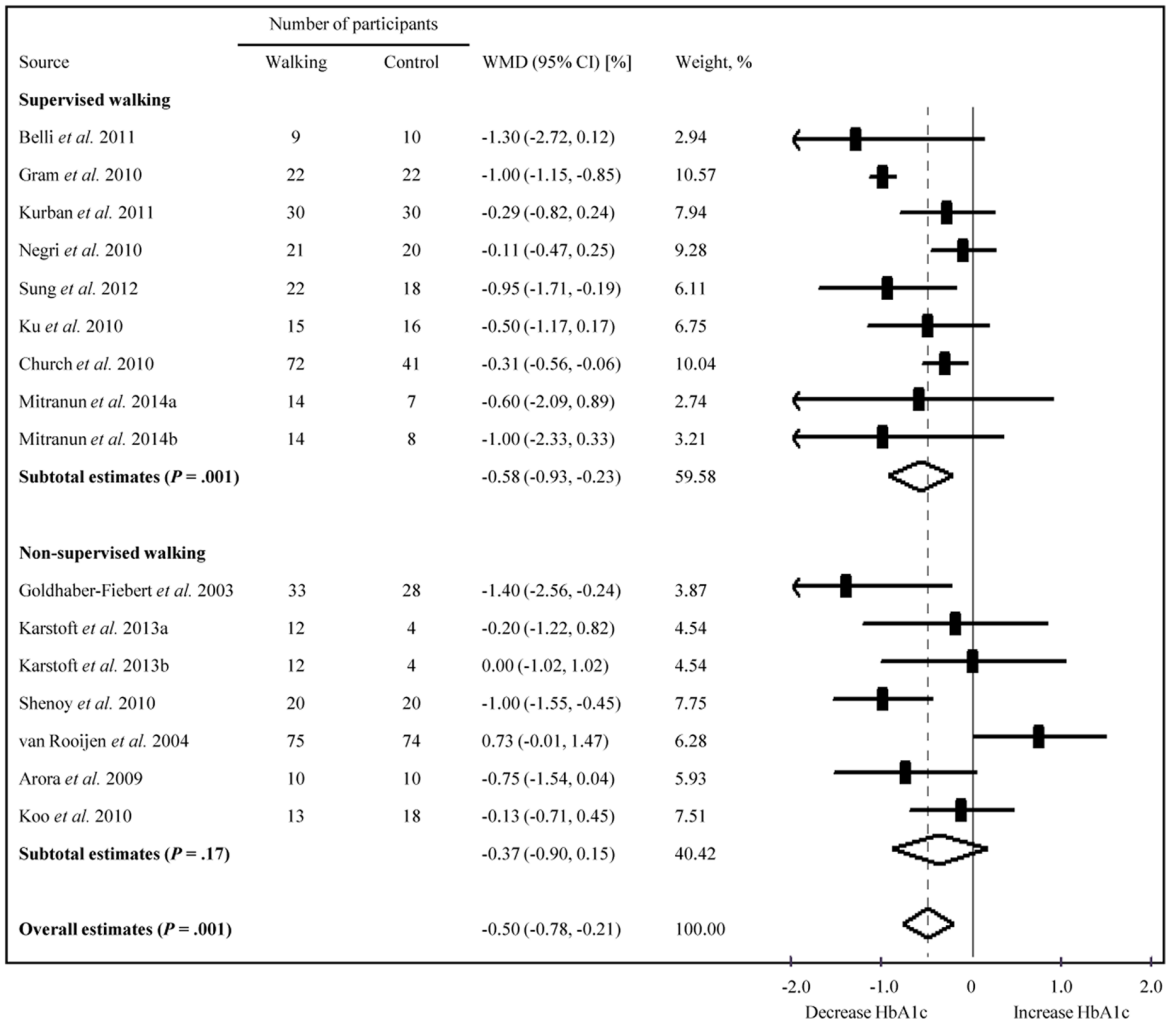
Forest plot of RCTs examining walking effects on HbA1c (%) in type 2 diabetes patients. RCTs, randomized controlled trials; HbA1c, glycosylated haemoglobin A1c; WMD, weighted mean difference; CI, confidence interval Summary estimates were analyzed with a random-effects model. HbA1c levels were converted from mmol/mol to % using the NGSP converter (available at http://www.ngsp.org/convert1.asp).

Sixteen trials involving 724 participants were included in the meta-analysis. Compared with the non-walking control, the pooled estimate showed a significant decrease in HbA1c (WMD −0.50%, 95% CI: −0.78% to −0.21%; Figure 2), but with a substantial heterogeneity between trials (*P*<.001, *I^2^* = 79.4%). Subgroup analyses showed supervised walking was associated with a significant decrease in HbA1c by 0.58% (9 trials, 391 participants; 95% CI: −0.93% to −0.23%), while the association between non-supervised walking and decreased HbA1c became statistically non-significant (7 trials, 333 participants; WMD −0.37%, 95% CI: −0.90% to 0.15%). Yet further subgroup analysis suggested that non-supervised walking with the use of motivational strategies was associated with a significant decrease in HbA1c (5 trials, 164 participants; WMD −0.53%, 95% CI: −1.05% to −0.02%), which made no difference from the supervised walking (*P* = .88). Univariate meta-regression analyses suggested that none of the covariates was the potential modifier of HbA1c change: baseline age (*β* coefficient, −0.90, *P* = .62), BMI (0.05, *P* = .33), sex (0.008, *P* = .14), duration of diabetes (−0.001, *P* = .98), walking frequency (0.16, *P* = .16), length of walking intervention (0.007, *P* = .71), or walking volume (0.001, *P* = .23). When individually removing each trial, pooled results were largely unchanged.

### Secondary outcomes

#### Effect on weight reduction

Sixteen trials involving 649 participants compared walking with the controls. The overall, pooled data showed that walking was associated with a significant reduction in BMI by 0.91 kg/m^2^ (95% CI: −1.22 to −0.59 kg/m^2^; Table 2). Heterogeneity among trials was negligible (*P* = .54, *I^2^*<1%). When trials were individually removed, pooled results remained largely unchanged.

**Table 2:** Effects of walking training on secondary outcomes.

Outcome	No. of studies (No. of subjects)	Effect size#		Heterogeneity	
		WMD (95% CI)	*P* value	*I^2^* (%)	*P* value
Weight reduction					
BMI, kg/m^2^	16 (649)	−0.91 (−1.22 to −0.59)	<.001	<1	= .54
Blood pressure, mmHg					
Systolic§	11 (497)	−1.69 (−5.22 to 1.85)	= .34	65.2	= .001
Diastolic	12 (509)	−1.97 (−3.94 to −0.0)	= .05	59.3	= .005
Lipoprotein, mmol/L					
HDL-C	9 (290)	0.02 (−0.06 to 0.10)	= .64	53.2	= .03
LDL-C	8 (270)	0.04 (−0.07 to 0.16)	= .49	7.0	= .38

WMD, weight mean difference; CI, confidence interval; BMI, body mass index; HDL-C, high-density lipoprotein cholesterol; LDL-C, low-density lipoprotein cholesterol.

SI conversion factors: to convert HDL-C and LDL-C from mg/dl to mmol/L, multiply by 0.0259.

# Effect size was calculated using a random-effects model.

§ The study of Karftoft *et al*. 2013a [20] was excluded because the baseline data of systolic blood pressure were not comparable between the intervention and control groups.

#### Effect on blood pressure

The overall, pooled estimates of trials reporting SBP (11 trials, 497 participants) showed a non-significant decrease in SBP among participants randomized to walking groups when compared with the controls (WMD −1.69 mmHg, 95% CI: −5.22 to 1.85 mmHg; *P* for heterogeneity = .001, *I^2^* = 65.2%; Table 2). The pooled estimates of trials reporting DBP (12 trials, 509 participants) showed a bigger reduction in DBP among participants randomized to walking groups than those randomized to non-walking groups (WMD −1.97 mmHg, 95% CI: −3.94 to −0.00 mmHg; *P* for heterogeneity = .005; *I^2^* = 59.3%; Table 2). Upon removal of trials individually from each meta-analysis, the overall WMD for DBP remained largely unchanged, while the WMD for SBP was changed to −3.20 mmHg (95% CI −5.35 to −1.05 mmHg) by removing the study by Gram *et al*. [19], a 16-week nordic walking intervention, which reported a larger increase in SBP (WMD 5.00 mmHg, 95% CI 2.67 to 7.33 mmHg) than any other trials.

#### Effect on lipoprotein profiles

Nine trials with 290 participants reported changes in HDL-C levels, and 8 trials with 270 participants reported changes in LDL-C levels. Walking training did not significantly increase the HDL-C levels (WMD 0.02 mmol/L, 95% CI: −0.06 to 0.10 mmol/L; *P* for heterogeneity = .03, *I^2^* = 53.2%; Table 2) or change the LDL-C levels (WMD 0.04 mmol/L, 95% CI: −0.07 to 0.16 mmol/L; *P* for heterogeneity = .38, *I^2^* = 7.0%; Table 2) among intervention participants. When trials were individually removed from each meta-analysis, pooled results regarding HDL-C or LDL-C were largely unchanged.

### Publication bias

There was no significant publication bias for all of the primary and secondary outcomes as evidenced by the Begg's test and Egger's test (all *P*>.05), except LDL-C (Egger’s test, *P* = .04).

## Discussion

The results of this meta-analysis suggest that in patients with type 2 diabetes, walking is associated with a significant decrease in HbA1c. The results further suggest that supervision is essential to walking training in decreasing HbA1c; while notably, the use of motivational strategies is also effective in decreasing HbA1c when performing non-supervised walking. The results also suggest that walking is associated with a reduced BMI and a lowered DBP. However, it shows inadequate evidence regarding the effects of walking in lowering SBP or altering lipoprotein levels in this meta-analysis.

Partly in line with our main results, a recent meta-analysis by Chudyk and Petrella [38] and another one by Snowling and Hopkins [39] demonstrated that aerobic exercise was associated with improved glycemic control in patients with type 2 diabetes. Yet it is worth noting that these meta-analyses included not only walking, but also other forms of aerobic exercise, such as bicycling or running. This could be prone to confounding when considering the possibility that physiological adaptations to exercise are specific to the stimulus applied [40], along with different physiological properties [41]. Since results from this meta-regression analysis did not find any modifier for HbA1c change, and given the common characteristics of the most trials, it is suggested to prescribe walking at a moderate intensity, 3–5 times/week, 120–150 minutes/week, for patients with type 2 diabetes to gain benefits on glycemic control.

Periodic supervision by qualified exercise trainers is recommended for patients with type 2 diabetes to ensure optimal glycemic control and to minimize injury risk when they undertake exercise training [6]. This recommendation has been further supported by a recently well-conducted meta-analysis [42], which noted that supervised exercise training was associated with a great decline in HbA1c level by 0.73% (95% CI: −1.06% to −0.40%). The observation from that meta-analysis is comparable to the results from our subgroup analyses, where a decrease in HbA1c by 0.58% (95% CI: −0.93% to −0.23%) was found. However, it should be acknowledged that full supervision by a qualified exercise trainer may not always be feasible for a considerable amount of patients with type 2 diabetes, given the high prevalence of type 2 diabetes [43] and the limited medical resources [36]. Despite that non-supervised walking was associated with a non-significant decrease in HbA1c, evidence from our further subgroup analyses suggested that even without supervision, walking with the use of motivational strategies would be helpful in reducing HbA1c, in which the magnitude of HbA1c reduction was comparable to that of supervised walking (*P* = .88). The motivational strategies utilized in this meta-analysis included peer support and the use of step counters, both of which have been proved to be effective in increasing daily movement (unstructured activity) [44], [45] and improving the training adherence or self-efficacy [44], [46]. It is likely that these related factors have contributed to the observed decrease in HbA1c, at least partly.

In addition to glycemic control, the meta-analysis showed that walking was associated with a significant reduction in BMI when compared with the non-walking controls. This result is consistent with the finding from Kelley *et al*., who reported a similar reduction in BMI from walking [15]. Since most patients with type 2 diabetes are overweight or obese, it therefore sounds reasonable to initially recommend walking to those patients to reduce body weight.

Snowling and Hopkins observed that aerobic exercise had minor effects in lowering SBP or DBP among patients with type 2 diabetes [39], although possibilities cannot be completely excluded that the overall effects would be masked by the different forms of aerobic exercise in that meta-analysis. In another meta-analysis, Kelley *et al*. noted that walking significantly reduced both SBP and DBP [13], yet this study enrolled sedentary adults other than patients with type 2 diabetes. Our meta-analysis showed that walking significantly lowered DBP but not SBP among patients with type 2 diabetes. In support of this, Murphy *et al*. showed that walking slightly reduced DBP but only had a tendency to lower SBP [14]. To some extent, this finding on DBP will help to prescribe walking to those type 2 diabetes patients who are especially vulnerable to elevated blood pressure [47]. This finding also provides evidence against the statement from the current guideline that reductions in DBP from aerobic exercise training are less common in patients with type 2 diabetes [6]. Our meta-analysis did not show a statistical support for the positive relationship between walking and increased HDL-C levels as well as decreased LDL-C levels among patients with type 2 diabetes. Partly in agreement with this, Kelley *et al*. found a non-significant increase in HDL-C associated with walking [15]. One possible explanation for this might be that all included trials had utilized walking training alone without weight reduction interventions, while the combination of both is more effective on lipids regulation [6], [48].

This meta-analysis has several strengths. First, it is to date the most comprehensive analysis that systematically and quantitatively assesses the beneficial effects of a particular form of aerobic exercise – walking, on glycemic control and several other cardiovascular risk factors among patients with type 2 diabetes. Second, it enriches the knowledge for prescribing walking to patients with type 2 diabetes, aiming to optimise glycemic control, weight reduction or DBP. Third, it was registered a priori (PROSPERO CRD42014009515) that minimised the selection and recall bias, and utilised strict inclusion and exclusion criteria.

This meta-analysis also has several limitations. First, as with any meta-analysis, the internal validity depends on the methodological quality of the included trials. Although all trials were judged with low risk of detection bias and described losses to follow-up, insufficient reporting of the randomization sequence generation and improper dealing with incomplete outcome data addressed in some of the trials reviewed would increase the risk of selection bias and attribution bias. It is recommended that future RCTs should describe adequate information about randomization and report data with guidelines. Second, despite no significant publication bias was detected by the Begg's test and Egger's test for each outcome variable except LDL-C, the risk of publication bias still cannot be fully ruled out due to the language restriction to English, the selection of only published papers, as well as the potentially underpowered statistical tests. Third, substantial heterogeneity of HbA1c was identified among the included trials, and it cannot be explained by a single related variable in the meta-regression analyses. Fourth, while our subgroup analyses showed that supervised walking was superior to non-supervised walking in decreasing HbA1c, it should be noted that these comparisons were indirect and somewhat less reliable when compared with the head-to-head trials. Therefore, future research with a head-to-head design is needed on this topic. Finally, because of the short-term walking intervention (only up to 6 months) reported in all the included trials except one with a 9-month intervention [30], results of this meta-analysis largely represent the short-term effects of walking intervention among patients with type 2 diabetes. Future RCTs that extend length of walking intervention are required.

## Conclusions

In conclusion, the meta-analysis shows that walking is associated with a decreased HbA1c among patients with type 2 diabetes. Supervision or the use of motivational strategies should be strongly recommended when prescribed walking to ensure optimal glycemic control. Walking is also effective in reducing BMI and lowering DBP, while evidence regarding its association with lowered SBP or improved lipoprotein profiles remains insufficient. Future RCTs with head-to-head designs comparing supervised walking versus non-supervised walking, and with extended length of walking interventions (>6 months), are required to strengthen the findings in this meta-analysis.

## Supporting Information

Table S1 **Search strategies.**
(DOCX)

Table S2 **PROSPERO CRD42014009515.**
(PDF)

Table S3 **Bias assessment of each randomized controlled trial.**
(DOC)

Checklist S1 **PRISMA Checklist.**
(DOC)
