# Supplementary material for: Impact of Walking on Glycemic Control and Other Cardiovascular Risk Factors in Type 2 Diabetes: A Meta-Analysis
Source: PLoS One. 2014 Oct 17;9(10):e109767. doi: 10.1371/journal.pone.0109767 (PMC4201471; doi:10.1371/journal.pone.0109767)
Supplement: Table S1 — Search strategies. (DOCX) [file pone.0109767.s001.docx]

**Table S1. Search strategies**

**Search strategy in PubMed**

#1 randomized controlled trial [Publication Type] OR controlled clinical trial [Publication Type] OR randomized [Title/Abstract] OR placebo [Title/Abstract] OR “clinical trials” [MeSH Major Topic] OR randomly [Title/Abstract] OR trial [Title]

#2 animals [MeSH Terms] NOT humans [MeSH Terms]

#3 #1 NOT #2

#4 "diabetes mellitus, type 2" [MeSH Terms] OR ketosis-resistant diabetes [Title/Abstract] OR ketosis resistant diabetes [Title/Abstract] OR maturity-onset diabetes [Title/Abstract] OR maturity onset diabetes [Title/Abstract] OR non insulin dependent diabetes [Title/Abstract] OR non-insulin-dependent diabetes [Title/Abstract] OR type 2 diabetes [Title/Abstract] OR diabetes mellitus type II [Title/Abstract] OR maturity onset diabetes mellitus [Title/Abstract] OR maturity onset diabetes mellitus [Title/Abstract] OR NIDDM [Title/Abstract] OR adult-onset diabetes mellitus [Title/Abstract] OR diabetes mellitus noninsulin dependent [Title/Abstract] OR type 2 diabetic [Title/Abstract]

#5 “physical activity” [MeSH Terms] OR exercise [MeSH Terms] OR walk [Title/Abstract] OR walking [Title/Abstract]

#6 #3 AND #4 AND #5

#7 #6 Filters: English, published from January 1, 1966 to August 8, 2014

**Search strategy in the Cochrane Central Register of Controlled Trials**

#1 andomized controlled trial [Publication Type] OR controlled clinical trial [Publication Type] OR randomized [Title/Abstract/Key Word] OR placebo [Title/Abstract/Key Word] OR randomly [Title/Abstract/Key Word] OR trial [Title/Abstract/Key Word] OR “clinical trial” [MeSH Terms]

#2 animals [MeSH Terms] NOT humans [MeSH Terms]

#3 #1 NOT #2

#4 "diabetes mellitus, type 2" [MeSH Terms] OR ketosis-resistant diabetes [Title/Abstract/Key Word] OR ketosis resistant diabetes [Title/Abstract/Key Word]OR maturity-onset diabetes [Title/Abstract/Key Word] OR maturity onset diabetes [Title/Abstract/Key Word] OR non insulin dependent diabetes [Title/Abstract/Key Word] OR non-insulin-dependent diabetes [Title/Abstract/Key Word] OR type 2 diabetes [Title/Abstract/Key Word] OR diabetes mellitus type II [Title/Abstract/Key Word] OR maturity onset diabetes mellitus [Title/Abstract/Key Word] OR maturity onset diabetes mellitus [Title/Abstract/Key Word] OR NIDDM [Title/Abstract/Key Word] OR adult-onset diabetes mellitus [Title/Abstract/Key Word] OR diabetes mellitus noninsulin dependent [Title/Abstract/Key Word] OR type 2 diabetic [Title/Abstract/Key Word]

#5 “motor activity” [MeSH Terms] OR exercise [MeSH Terms] OR walk [Title/Abstract/Key Word] OR walking [MeSH Terms]

#6 #3 AND #4 AND #5

#7 #6 Filters: published from January 1, 1990 to August 8, 2014 (by hand)

**Search strategy in Web of SCI**

#1 diabet* [Title] OR type 2 diabetes [Topic] OR “diabetes mellitus, type 2” [Topic]

#2 exercise [Title] OR training [Title] OR physical activity [Title] OR walk [Title] OR walking [Title]

#3 random* [Topic] OR trial [Topic]

#4 animals [Topic] NOT humans [Topic]

#5 #3 NOT #4

#6 #1 AND #2 AND #5

#7 #6 Filters: English [Language], Article [Document Type]

#8 #7 published from January 1, 1945 to August 8, 2014 (by hand)
