# Supplementary material for: Impact of Walking on Glycemic Control and Other Cardiovascular Risk Factors in Type 2 Diabetes: A Meta-Analysis
Source: PLoS One. 2014 Oct 17;9(10):e109767. doi: 10.1371/journal.pone.0109767 (PMC4201471; doi:10.1371/journal.pone.0109767)
Supplement: Table S2 — PROSPERO CRD42014009515. (PDF) [file pone.0109767.s002.pdf]

PROSPERO International prospective register of systematic reviews

Review title and timescale

1 Review title
Give the working title of the review. This must be in English. Ideally it should state succinctly the interventions or exposures being reviewed and the associated health or social problem being addressed in the review.
Impact of walking on glycemic control and other cardiovascular risk factors in type 2 diabetes: a meta-analysis

2 Original language title
For reviews in languages other than English, this field should be used to enter the title in the language of the review. This will be displayed together with the English language title.

3 Anticipated or actual start date
Give the date when the systematic review commenced, or is expected to commence.
25/04/2014

4 Anticipated completion date
Give the date by which the review is expected to be completed.
30/06/2014

5 Stage of review at time of this submission
Indicate the stage of progress of the review by ticking the relevant boxes. Reviews that have progressed beyond the point of completing data extraction at the time of initial registration are not eligible for inclusion in PROSPERO. This field should be updated when any amendments are made to a published record.

The review has not yet started ×

Review stage	Started	Completed
Preliminary searches	No	Yes
Piloting of the study selection process	No	Yes
Formal screening of search results against eligibility criteria	No	Yes
Data extraction	No	Yes
Risk of bias (quality) assessment	No	Yes
Data analysis	No	Yes

Provide any other relevant information about the stage of the review here.

Review team details

6 Named contact
The named contact acts as the guarantor for the accuracy of the information presented in the register record.
Shanhu Qiu

7 Named contact email
Enter the electronic mail address of the named contact.
tigershanhu@126.com

8 Named contact address
Enter the full postal address for the named contact.
Xinmofan Road No.3, Nanjing, China

9 Named contact phone number
Enter the telephone number for the named contact, including international dialing code.
0086 025 83285150

10 Organisational affiliation of the review
Full title of the organisational affiliations for this review, and website address if available. This field may be completed as 'None' if the review is not affiliated to any organisation.
Zhongda Hospital, Southeast University, China

Website address:
http://www.njzdyy.com

11 Review team members and their organisational affiliations

Give the title, first name and last name of all members of the team working directly on the review. Give the organisational affiliations of each member of the review team.

Title	First name	Last name	Affiliation
Dr	Shanhu	Qiu	Department of Endocrinology, Zhongda Hospital, Institute of Diabetes, Medical School, Southeast University
Mrs	Xue	Cai	Department of Endocrinology, Zhongda Hospital, Institute of Diabetes, Medical School, Southeast University
Professor	Zilin	Sun	Department of Endocrinology, Zhongda Hospital, Institute of Diabetes, Medical School, Southeast University
Professor	Jürgen Michael	Steinacker	Department of Medicine II, Section of Sports and Rehabilitation Medicine, Ulm University

12 Funding sources/sponsors

Give details of the individuals, organizations, groups or other legal entities who take responsibility for initiating, managing, sponsoring and/or financing the review. Any unique identification numbers assigned to the review by the individuals or bodies listed should be included.

No funding or sponsors

13 Conflicts of interest

List any conditions that could lead to actual or perceived undue influence on judgements concerning the main topic investigated in the review.

Are there any actual or potential conflicts of interest?

None known

14 Collaborators

Give the name, affiliation and role of any individuals or organisations who are working on the review but who are not listed as review team members.

Title	First name	Last name	Organisation details
Dr	Uwe	Schumann	Department of Medicine II, Section of Sports and Rehabilitation Medicine, Ulm University
Dr	Martina	Velders	Department of Medicine II, Section of Sports and Rehabilitation Medicine, Ulm University

Review methods

15 Review question(s)

State the question(s) to be addressed / review objectives. Please complete a separate box for each question.

Although meta-analyses showed health benefits of aerobic exercise on cardiovascular risk factors among type 2 diabetes patients, they failed to specify the actual effects of a particular exercise, such as walking. Besides, inconsistent results have been shown regarding the effects of walking training on cardiovascular risk factors in patients with type 2 diabetes. Therefore, we aim to examine the association of walking with clinical markers of cardiovascular risk, such as glycaemic control, weight reduction, blood pressure, and lipoprotein profiles, among patients with type 2 diabetes by meta-analyzing randomized controlled trials.

16 Searches

Give details of the sources to be searched, and any restrictions (e.g. language or publication period). The full search strategy is not required, but may be supplied as a link or attachment.

Primary articles were sought in the following databases: PubMed (January 1, 1966 to August 8, 2014), Web of Science (January 1, 1945 to August 8, 2014) and the Cochrane Central Register of Controlled Trials (CENTRAL) (January 1, 1966 to August 8, 2014). The initial computer-based search strategies comprised common text words and

Medical Subject Heading terms related to exercise, walking and type 2 diabetes, as well as entry terms associated with a highly sensitive search filter for RCTs. Searches were limited to human beings and the language was restricted to English.

17 URL to search strategy

If you have one, give the link to your search strategy here. Alternatively you can e-mail this to PROSPERO and we will store and link to it.

I give permission for this file to be made publicly available
Yes

18 Condition or domain being studied

Give a short description of the disease, condition or healthcare domain being studied. This could include health and wellbeing outcomes.
The effects of walking on glycemic control and other cardiovascular risk factors in patients with type 2 diabetes.

19 Participants/population

Give summary criteria for the participants or populations being studied by the review. The preferred format includes details of both inclusion and exclusion criteria.
Inclusion: patients diagnosed with type 2 diabetes. Exclusion: patients diagnosed with type 1 diabetes, pre-diabetes, or gestational diabetes.

20 Intervention(s), exposure(s)

Give full and clear descriptions of the nature of the interventions or the exposures to be reviewed
Inclusion: engaged in a structured walking training programme with or without health education lasting at least 8 weeks. Exclusion: had multiple exercise interventions (i.e., combined/mixed with other types of exercise), or interventions consisted only of recommending increased daily walking steps by motivational tools, or combined with dietary intervention.

21 Comparator(s)/control

Where relevant, give details of the alternatives against which the main subject/topic of the review will be compared (e.g. another intervention or a non-exposed control group).
Inclusion: compared with control groups which received usual care without regular exercise training, or just asked to maintain their normal lifestyle. Exclusion: compared with control groups receiving regular exercise training.

22 Types of study to be included initially

Give details of the study designs to be included in the review. If there are no restrictions on the types of study design eligible for inclusion, this should be stated.
Inclusion: had a RCT study design. Exclusion: were non-randomized studies, posters or just abstracts.

23 Context

Give summary details of the setting and other relevant characteristics which help define the inclusion or exclusion criteria.

24 Primary outcome(s)

Give the most important outcomes.
Glycaemic control as assessed by HbA1c

Give information on timing and effect measures, as appropriate.
Fasting blood samples were measured with standard procedures for clinical markers.

25 Secondary outcomes

List any additional outcomes that will be addressed. If there are no secondary outcomes enter None.
Weight reduction as measured by body mass index (BMI), blood pressure as indicated by diastolic blood pressure (DBP) and systolic blood pressure (SBP), and lipoprotein profiles as represented by HDL-C and LDL-C

Give information on timing and effect measures, as appropriate.
blood pressure was measured at rest (after completing the walking programme), lipoprotein profiles were measured with standard procedures using fasting blood samples.

26 Data extraction, (selection and coding)

Give the procedure for selecting studies for the review and extracting data, including the number of researchers involved and how discrepancies will be resolved. List the data to be extracted.

27 Risk of bias (quality) assessment

State whether and how risk of bias will be assessed, how the quality of individual studies will be assessed, and whether and how this will influence the planned synthesis.

The methodological quality of each eligible study will be assessed in accordance with the Cochrane Collaboration's 'Risk of Bias' Tool, including random sequence generation, allocation concealment, blinding of participants and personnel, blinding of outcome assessment, incomplete outcome data and selective reporting.

28 Strategy for data synthesis

Give the planned general approach to be used, for example whether the data to be used will be aggregate or at the level of individual participants, and whether a quantitative or narrative (descriptive) synthesis is planned. Where appropriate a brief outline of analytic approach should be given.

For studies that reported standard error of the mean, the standard deviation (SD) will be obtained by multiplying by the square root of the corresponding sample size. For studies that compared two different walking interventions with a single control group, the "shared" group will be split into two different groups with weighted smaller sample sizes in relation to different walking interventions. This will be applied to give reasonably independent comparisons and overcome a unit-of-analysis error. For studies that gave outcomes at more than one time point during the intervention, data from the latest time point will be used for primary analyses. Change scores from baseline or final values of each outcome variable will be entered in the same meta-analysis, as suggested in the Cochrane Handbook for Systematic Reviews. Cochran Q test and I-squared test will be used to assess the heterogeneity. To account for between-study heterogeneity, the pooled-effect estimates expressed by weighted mean difference (WMD) and the corresponding 95% confidence intervals (CIs) of each outcome will be calculated by a random-effects model.

29 Analysis of subgroups or subsets

Give any planned exploration of subgroups or subsets within the review. 'None planned' is a valid response if no subgroup analyses are planned.

Subgroup analyses will be performed to investigate the differences in outcome effects across studies on the basis of supervision given status (with or without).

Review general information

30 Type of review

Select the type of review from the drop down list.

Intervention

31 Language

Select the language(s) in which the review is being written and will be made available, from the drop down list. Use the control key to select more than one language.

English

Will a summary/abstract be made available in English?

Yes

32 Country

Select the country in which the review is being carried out from the drop down list. For multi-national collaborations select all the countries involved. Use the control key to select more than one country.

China, Germany

33 Other registration details

List places where the systematic review title or protocol is registered (such as with he Campbell Collaboration, or The Joanna Briggs Institute). The name of the organisation and any unique identification number assigned to the review by that organization should be included.

34 Reference and/or URL for published protocol

Give the citation for the published protocol, if there is one.

Give the link to the published protocol, if there is one. This may be to an external site or to a protocol deposited with CRD in pdf format.

I give permission for this file to be made publicly available
Yes

35 Dissemination plans
Give brief details of plans for communicating essential messages from the review to the appropriate audiences.
Do you intend to publish the review on completion?
Yes

36 Keywords
Give words or phrases that best describe the review. (One word per box, create a new box for each term)
walking

cardiovascular risk

type 2 diabetes

meta-analysis

37 Details of any existing review of the same topic by the same authors
Give details of earlier versions of the systematic review if an update of an existing review is being registered, including full bibliographic reference if possible.

38 Current review status
Review status should be updated when the review is completed and when it is published.
Ongoing

39 Any additional information
Provide any further information the review team consider relevant to the registration of the review.

40 Details of final report/publication(s)
This field should be left empty until details of the completed review are available.
Give the full citation for the final report or publication of the systematic review.
Give the URL where available.
