# Supplementary material for: Impact of Walking on Glycemic Control and Other Cardiovascular Risk Factors in Type 2 Diabetes: A Meta-Analysis
Source: PLoS One. 2014 Oct 17;9(10):e109767. doi: 10.1371/journal.pone.0109767 (PMC4201471; doi:10.1371/journal.pone.0109767)
Supplement: Table S3 — Bias assessment of each randomized controlled trial. (DOC) [file pone.0109767.s003.doc]

# Table S3. Bias assessment of each randomized controlled trial (RCT)^#^

| Author, year | Random sequence generation | Allocation concealment | Blinding of participants and personnel^§^ | Blinding of outcome assessment | Incomplete outcome data addressed | Selective reporting |
| --- | --- | --- | --- | --- | --- | --- |
| Belli *et al*. 2011 | Low | Unclear | High | Low | High | Low |
| Goldhaber-Fiebert *et al*. 2003 | Low | High | High | Low | Low | Low |
| Gram *et al*. 2010 | Low | Unclear | High | Low | Low | Low |
| Karstoft *et al*. 2013a^*^ | Low | Unclear | High | Low | Low | Low |
| Karstoft *et al*. 2013b^*^ | Low | Unclear | High | Low | Low | Low |
| Kurban *et al*. 2011 | Unclear | Unclear | High | Low | Low | Low |
| Negri *et al*. 2010 | Low | High | High | Low | High | Low |
| Shenoy *et al*. 2010 | Low | Unclear | High | Low | Low | Low |
| Sung *et al*. 2012 | Low | Unclear | High | Low | Unclear | Low |
| van Rooijen *et al*. 2004 | Low | Low | High | Low | Low | Low |
| Ku *et al*. 2010 | Low | Unclear | High | Low | Low | Low |
| Arora *et al.* 2009 | Unclear | Unclear | High | Low | Low | Low |
| Moghadasi *et al*. 2013 | Unclear | Unclear | High | Low | Low | Low |
| Kaplan *et al*. 1985 | Unclear | Unclear | High | Low | Low | Low |
| Church *et al*. 2010 | Low | Low | High | Low | Low | Low |
| Dixit *et al*. 2014 | Low | Unclear | High | Low | High | Low |
| Koo *et al.* 2010 | Unclear | Unclear | High | Low | Low | Low |
| Kwon *et al*. 2010 | Unclear | Unclear | High | Low | High | Low |
| Mitranun *et al*. 2014a^**^ | Unclear | Unclear | High | Low | Low | Low |
| Mitranun *et al*. 2014b^**^ | Unclear | Unclear | High | Low | Low | Low |

^#^ Summary assessments of the risk of bias for each RCT within studies: Low risk of bias, low risk of bias for all key domains; Unclear risk of bias, unclear risk of bias for one or more key domains; High risk of bias, high risk of bias for one or more key domains.

^§^ Because of the nature of exercise intervention, complete blinding of participants and personnel is impossible for each RCT.

^*^ The same study which included 2 different walking groups: “a” was a continuous walking training group; “b” was an energy expenditure–matched interval-walking training group.

^**^ The same study which included 2 different walking groups: “a” was a continuous walking training group; “b” was a total oxygen consumption-matched interval-walking training group.
